# Long-term optical stimulation of channelrhodopsin-expressing neurons to study network plasticity

**DOI:** 10.3389/fnmol.2013.00022

**Published:** 2013-08-20

**Authors:** Gabriele Lignani, Enrico Ferrea, Francesco Difato, Jessica Amarù, Eleonora Ferroni, Eleonora Lugarà, Stefano Espinoza, Raul R. Gainetdinov, Pietro Baldelli, Fabio Benfenati

**Affiliations:** ^1^Department of Neuroscience and Brain Technologies, Istituto Italiano di TecnologiaGenoa, Italy; ^2^Department of Experimental Medicine, University of GenoaGenoa, Italy

**Keywords:** long-term recordings, primary neurons, optogenetics, network activity, network plasticity

## Abstract

Neuronal plasticity produces changes in excitability, synaptic transmission, and network architecture in response to external stimuli. Network adaptation to environmental conditions takes place in time scales ranging from few seconds to days, and modulates the entire network dynamics. To study the network response to defined long-term experimental protocols, we setup a system that combines optical and electrophysiological tools embedded in a cell incubator. Primary hippocampal neurons transduced with lentiviruses expressing channelrhodopsin-2/H134R were subjected to various photostimulation protocols in a time window in the order of days. To monitor the effects of light-induced gating of network activity, stimulated transduced neurons were simultaneously recorded using multi-electrode arrays (MEAs). The developed experimental model allows discerning short-term, long-lasting, and adaptive plasticity responses of the same neuronal network to distinct stimulation frequencies applied over different temporal windows.

## Introduction

A single neuron conveys an array of signals to the neural circuit in which it operates by expressing several forms of plasticity (Daoudal and Debanne, 2003; Fioravante and Regehr, 2011). The neural units undergo a variety of activity-dependent changes that affect not only the synaptic machinery, but also the intrinsic firing properties of the neuron and promote structural rearrangements of its axonal and dendritic trees (Turrigiano, 2008; Grubb and Burrone, 2010; Feldman, 2012; Remme and Wadman, 2012). Neural networks store information through multiple processes of activity-dependent plasticity that differently affects various populations of neurons (Chang et al., 2010; Lau and Murthy, 2012; Shin et al., 2012).

Acting on a millisecond-to-minute timescale, short-term plasticity (STP) is believed to play an important role in synaptic computation and network information processing (Abbott and Regehr, 2004; Deng and Klyachko, 2011). On the other hand, persistent forms of activity-dependent plasticity occurring in neural ensembles and involving long-term changes in membrane excitability and synaptic transmission, provide a physiological substrate for learning and long-term memory storage (Caroni et al., 2012).

Finally, homeostatic processes have been proposed to explain the discrepancy between the high dynamics in brain circuit plasticity and the stability of brain function. Homeostatic plasticity works as a slow process that modifies both synaptic transmission efficacy and membrane excitability, keeping the firing rate of neural circuits within a physiological range (Turrigiano, 2008). Together with other processes, such as conventional short- and long-term plasticity, homeostatic plasticity changes the patterns of activity of a network, ensuring a stable and input-dependent functional connectivity (Turrigiano, 2012).

In this scenario, the effects of a signal transmitted through a defined brain circuit can vary enormously depending on the recent history of the network and these variations can last from milliseconds to months. To study these multiple forms of plasticity operating in an extremely wide temporal scale, we designed and developed a simple and low-cost set-up that allows controlling and maintaining the environmental parameters during long-term electrophysiological measurements with multi-electrode array (MEA).

The non-invasive measurement conditions of MEA devices allows long-term recording of the activity of dissociated neuronal networks as well as physical and bidirectional interfacing of the networks with the external world (Tessadori et al., 2012; Zullo et al., 2012). However, it is necessary to establish stable physiological conditions of neuronal cultures to avoid artifactual activity fluctuations and/or cellular dysfunctions. The major critical task is constituted by the MEA amplification system that suffers the high humidity levels (90%) required for culturing cells, and thus the majority of MEA recordings are conducted outside the incubator under normal ambient conditions (Chiappalone et al., 2008; Bologna et al., 2010). Such approach limits the duration of the experimental sessions to a couple of hours (Potter and Demarse, 2001; Biffi et al., 2012), and are not suited for long-term studies because data reproducibility might be affected over time. To overcome these problems, several laboratories developed various solutions, in order to perform long-lasting recording under controlled environmental conditions, i.e., with constant temperature, CO_2_, and humidity levels (Gross and Schwalm, 1994; Blau and Ziegler, 2001; Pancrazio et al., 2003; Kim et al., 2012; Yang et al., 2012). Indeed, these solutions allowed investigating neuronal adaptation to repeated external stimuli during neuronal development. Recently, it has been shown that long-term optogenetic stimulation administered to neurons kept in the incubator elicits homeostatic changes in neuronal morphology at the single cell level (Goold and Nicoll, 2010; Grubb and Burrone, 2010). Although optogenetic stimulation has been already integrated with MEA devices(Takahashi et al., 2012; Dranias et al., 2013), the potentiality of these low invasive methods was not fully exploited to characterize network activity in long-lasting experiments in the presence of chronic stimulation triggering adaptive network responses over time.

In this paper, we combined long-term optogenetic stimulations with MEA recordings in a controlled *in vitro* environment to monitor functional rearrangements of the neuronal network. Infection of the entire neuronal network with the algal protein Channelrhodopsin-2/H134R (ChR2/H134R) (Lin et al., 2009; Lin, 2011) allowed to optically control neuronal excitability through a minimally invasive and temporally precise stimulation that could potentially be genetically targeted to specific neuronal sub-populations (i.e., excitatory vs. inhibitory neurons) or specific subcellular domains. This approach allows inducing, detecting and tracking changes in spontaneous and evoked neuronal networks activity while they adapt to various optical stimuli.

## Materials and methods

### Cell cultures

Hippocampal cultures were prepared from mouse C57BL/6J (E17-E18) embryos as previously described (Baldelli et al., 2007). All experiments were carried out in accordance with the guidelines of the European Community Council (Directive 2010/63/EU of September 22nd, 2010) and were approved by the Italian Ministry of Health. Dissociated hippocampal and cortical neurons were plated at 200 cells/mm^2^ on coverslips or MEA coated with Poly-D-Lysine (0.1 mg/ml) and maintained in Neurobasal medium containing B27 Supplement and Glutamax (Invitrogen, Monza, Italy).

### Patch-clamp recordings

Action potential activation was studied by whole-cell current-clamp recordings, and ChR2/H134R-induced current by voltage-clamp recordings. Both approaches were performed using a Multiclamp 700B amplifier (Axon Instruments, Molecular Devices, Sunnyvale CA, USA) using an upright BX51WI microscope (Olympus, Japan) equipped with Nomarski optics. The age of the patched neurons ranged between 15 and 19 days *in vitro* (div). Patch electrodes, fabricated from thick borosilicate glasses, were pulled and fire-polished to a final resistance of 5–7 MΩ. Experiments were performed at 22–24°C. All experiments were recorded in Tyrode extracellular solution to which D-(-)-2-amino-5-phosphonopentanoic acid (D-AP5; 50 μM), 6-cyano-7-nitroquinoxaline-2,3-dione (CNQX; 10 μM) and bicuculline methiodide (30 μM) were added to block NMDA, non-NMDA and GABA_A_ receptors, respectively. The internal solution (K gluconate) contained (in mM) 126 K gluconate, 4 NaCl, 1 MgSO_4_, 0.02 CaCl_2_, 0.1 BAPTA, 15 Glucose, 5 Hepes, 3 ATP, 0.1 GTP, pH 7.3. Only cells with resting membrane potentials between −55 and −70 mV, access resistance <10 MΩ and leak current <100 pA were considered for analysis. Current-clamp recordings were made at resting membrane potential, and action potential firing was induced by light pulses of 1, 5, or 10 ms. Voltage-clamp recordings were performed at −70 mV and inward current was induced by light pulses of 500 and 1 ms. Voltage traces were acquired at 5 kHz and low-pass filtered at 2.5 kHz.

### Virus production and infection

All experiments were performed using a pLenti-Synapsin-hChR2(H134R)-EYFP-WPRE. The plasmid was a kind gift of Karl Deisseroth (Stanford University, California, USA). Third-generation LVs were produced by transient four-plasmid co-transfection into HEK293T cells using the calcium phosphate transfection method. Supernatants were collected, passed through a 0.45 μm filter and purified by ultracentrifugation as previously described (De Palma and Naldini, 2002). Viral vectors were titrated at concentrations ranging from 1 × 10^8^ to 5 × 10^9^ transducing units/ml. Cultures were infected at 8–12 div by using 2–5 multiplicity of infection, and neurons were checked for positive transduction at 15–19 div. The efficiency of transduction, estimated by counting neurons expressing EYFP protein respect to the total number of DAPI-stained cells, was > 90%.

### Immunofluorescence

Primary hippocampal neurons were fixed in 4% paraformaldehyde, 4% sucrose in 0.12 M phosphate buffer, pH 7.4, rinsed several times in phosphate-buffered saline (PBS), blocked and permeabilized in 0.1% gelatin/0.3% Triton X-100 in PBS. Samples were sequentially incubated with an anti-GFP primary antibody (A11122, Invitrogen) and a 488-fluorochrome-conjugated secondary antibody (Invitrogen). After several washes in PBS, coverslips were mounted using Prolong Gold anti-fade reagent with DAPI (Invitrogen). Images were acquired using a 40 × objective in a Leica SP5 confocal microscope.

### MEA recordings

Dissociated hippocampal neurons were plated onto a planar Muse MEA (M64-GL1-30Pt200, Axion Biosystems, Atlanta, GA). The electrode diameter was 30 μm and the orthogonal distances between electrodes were 200 μm. The Muse 64 channel amplifier connected to an external hardware controller via a National Instrument analog-to-digital card was used to amplify extracellular signals. Raw data were digitized at 20 kHz and stored on a hard disk for off-line analysis. Spike detection of single extracellular action potentials was performed using the Axion Biosystem software using a voltage threshold 6–7-fold the standard deviation of the noise over 200 Hz high-pass filtered traces. Spike train data were analyzed using Neuroexplorer software (Plexon, Dallas, TX) to compute the mean firing rate. In all experiments, mean firing rates were normalized to the respective baseline, after a stabilization period of 2 h. Cultures kept in the incubator system were recorded between 18 and 21 div under control conditions or during the administration of various photo-stimulation protocols.

### Photo-stimulation protocols

To induce long-term plasticity in neuronal cultures, we used a tetanic stimulation protocol consisting of a 20–40 Hz train lasting 1 s and repeated every 10 s for 10 min. To evaluate adaptive plasticity, a chronic photo-stimulation protocol consisting of 125 ms tetanic stimulation at 40 Hz was repeated every minute for 48 h. In all protocols, the light-pulse stimulus with the 470 nm LED was lasting 10 ms with a power of 0.5 mW/mm^2^. To evaluate the response of the cultures to the sustained photostimulation, firing rate data were averaged in 12 h epochs. Then, the average baseline firing rate (determined over the 24 h prior to stimulation) was subtracted from the mean firing rate (MFR) of the respective epoch and the resulting value was normalized to the peak firing rate of ChR2/H134R-infected cultures as follows:

$${\text{MFR}}_{\text{norm}}=\frac{\left({\text{MFR}}_{\text{epoch }(\text{x})}-{\text{MFR}}_{\text{baseline}}\right)}{\text{Peak }{\text{MFR}}_{\text{ChR2}/\text{H134R}}}*100$$

### Viability assays

Glutamate released from cultured hippocampal neurons was measured using an Amplex Red glutamic acid/glutamate oxidase assay kit (Invitrogen). Briefly, samples were collected after baseline and after stimulation. Then, the medium was collected and analyzed for glutamate concentration according to the manufacturer's instructions. The resulting increase in fluorescence over time was measured with the use of a fluorescence microplate reader (Tecan Infinite 500) with excitation at 538 nm and emission at 594 nm. Cell culture viability was evaluated with propidium iodide and Hoechst-33342 (all from Sigma, Milan, Italy) as previously described (Frega et al., 2012). The cell-impermeable red fluorescent dye propidium iodide enters only dead cells in which cell membrane integrity is compromised, while the cell-permeable blue nuclear dye Hoechst-33342 is used to visualize the total number of cells.

### Statistical analysis

Data are expressed as means ± s.e.m. for number of cells or of independent network preparations (n). Data were analyzed using non-parametric tests (Mann-Whitney test, Friedman's test for repeated measures, Friedman's Two-Way ANOVA by Ranks, Kruskal–Wallis test and the Dunn's *post-hoc* multiple comparison test) using the SPSS software (SPSS, Inc., Chicago, IL). The significance level was preset to *p* < 0.05.

## Results

### Build-up of the opto/MEA chronic stimulation/recording setup

**Figure 1:**
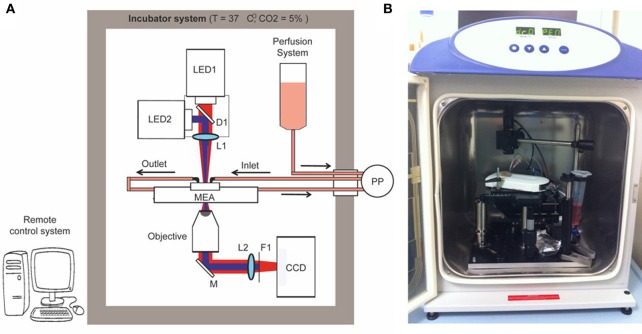
**Optical setup.** Schematic overview **(A)** and picture **(B)** of the OM-CSR setup combining optical recordings, optical stimulation and MEA recordings embedded in an incubator that maintains a physiological environment for neuronal cultures. LED1/2, light emitted diodes (470 and 530 nm); M, mirror; D, dichroic; L, lens; F, filter; PP, peristaltic pump.

We constructed a simple optical microscope to simultaneously perform long-term optical stimulation and imaging of neural networks cultured on MEA chips (opto/MEA chronic stimulation/recording (OM-CSR) system; Figure 1). The light sources of the optical setup were two LEDs (M735L3 and M470L2 equipped with two LEDD1B controllers—Thorlabs): one emitting at 735 nm wavelength to reduce phototoxicity during bright-field optical imaging (LED1), and the other emitting at 470 nm to stimulate neuronal network transduced with ChR2/H134R (LED2). The light from the two LEDs was combined through the dichroic mirror D1 (FF495-DI02—Semrock) and focused on the sample by the lens L1 (30 mm focal length VIS doublets—Thorlabs), to produce a homogeneous circular illumination spot of 4 mm diameter, which covers the entire active area of the MEA chip. The light collected with an air-immersion microscope objective was reflected from M1 (PF10-03-P01—Thorlabs) and focused on the CCD camera by L2 (100 mm focal length VIS doublets—Thorlabs). Only the infrared portion (IR) of the light passed through the filter F1 (BLP01-594R-25—Semrock) and reached the CCD camera (BASLER-Pilot 04-PIA1000-48GM—ATvision) for imaging. The MEA amplifier and the primary neurons grown onto the MEA chip were positioned on a manual stage. To perform imaging of the entire network topography during long-term photo-stimulation and electrophysiological recordings, a 10 × magnification objective (RMS10X Plan Achromat Objective, 0.25 NA—Olympus) was used.

To maintain physiological parameters of the neuronal culture within a narrow range (5% CO_2_ and 37°C), the entire optical system was embedded in an incubator system (Galaxy 48-R Incubator—EuroClone). The use of a CCD camera instead of a classical eyepiece system reduces the dimensions of the optical setup within the small incubator and allows monitoring the morphology of the network in remote. We set 0% humidity in the incubator system to preserve the performance of the optical/electronic components. However, to minimize and compensate medium evaporation and thus stabilize the osmolarity, the MEA chip was closed with a custom-designed polydimethylsiloxane (PDMS) lid (Blau et al., 2009), and a perfusion system controlled by an external peristaltic pump (PP; REGLO Digital MS-2/8—ISMATEC) fed the culture at a flow rate of 8 μ l/min.

A custom-made software interface based on LabVIEW (National Instruments) generated TTL-synchronization signals sent through a D/A USB-board (USB-6008-NI-DAQmx—TESEO) to the various devices (LED1, LED2, CCD), allowing remote control of the experimental protocol. To perform live imaging, the IR light pulses were synchronized with the CCD image acquisition (500 ms light-pulse duration every 5 min, synchronized with a CCD exposure time of 1 s). To perform photostimulation, an oscilloscope produced a square wave TTL-signal (0–5 V signal amplitude, 20–40 Hz repetition rate and 20–40% duty cycle). The waveform coming from the oscilloscope was time-gated by a passive-relay-driven by a TTL-signal from the D/A USB-board.

### Inward currents and action potential could be reliably induced in ChR2/H134R-expressing neurons

**Figure 2:**
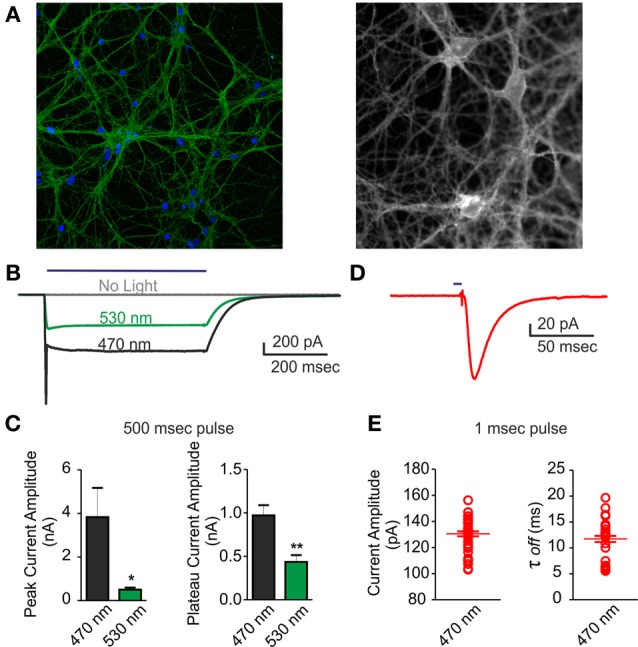
**Voltage-clamp characterization of ChR2/H134R-expressing neurons.** An inward current could be triggered by illumination with the 470 nm LED in neurons infected with ChR2/H134R. **(A)** (Left) Confocal image of 18 div neurons infected with ChR2/H134R-YFP labeled with DAPI (blue) and anti-GFP (green) and (*right*) live imaging of infected neurons at 18 div during electrophysiological recordings (fluorescent signal: ChR2-YFP). **(B)** Representative traces of inward currents induced by a 500 ms light pulse at either 470 (black) or 530 nm (green) wavelength or without illumination (gray). **(C)** Mean (±s.e.m.) peak amplitude and plateau amplitude of inward current induced by a 500 ms light pulse at 470 or 530 nm (*n* = 6). **(D)** Representative trace of an inward current induced by 1 ms light pulse at 470 nm. **(E)** Mean (±s.e.m.) current amplitude and decay (τ_off_) of inward current induced by 1 ms light pulse at 470 nm (*n* = 45 from 6 independent experiments). The individual data are also shown in the plot. ^*^p < 0.05; ^**^p < 0.001 Mann–Whitney test vs. 470 nm.

A good performance of photo-stimulation is due to the extent of ChR2/H134R expression and membrane targeting, as well as to a strict control of LED power and wavelength. To ascertain that action potentials could be evoked by light in most of neurons under our conditions during long-term MEA recordings, we performed patch-clamp experiments on the same cultures in both voltage- and current-clamp configurations. First, we acquired confocal images and showed that over 90% of neurons infected with the ChR2/H134R lentiviral vector expressed the protein (Figure 2A). After setting LED light power to a level previously described as capable to generate a large inward current (0.5 mW/mm^2^) (Lin et al., 2009; Gunaydin et al., 2010), we performed a complete electrophysiological characterization by stimulating neurons with a 500 ms light pulse at either 470 or 530 nm in voltage-clamp configuration. As expected, both the peak and the plateau current amplitude were larger at 470 nm than at 530 nm (peak: 470 nm = 3.84 ± 1.32 nA, 530 nm = 0.51 ± 0.08 nA; plateau: 470 nm = 0.98 ± 0.11 nA, 530 nm = 0.43 ± 0.07 nA) (Figures 2B,C). Moreover, we were able to induce an inward current with 1 ms light pulse with the 470 nm LED (Figures 2D,E) demonstrating that current amplitude and channel τ-off were stable and reproducible among independent experiments (amplitude: 130.5 ± 1.82 pA; τ-off: 11.73 ± 0.59 ms).

**Figure 3:**
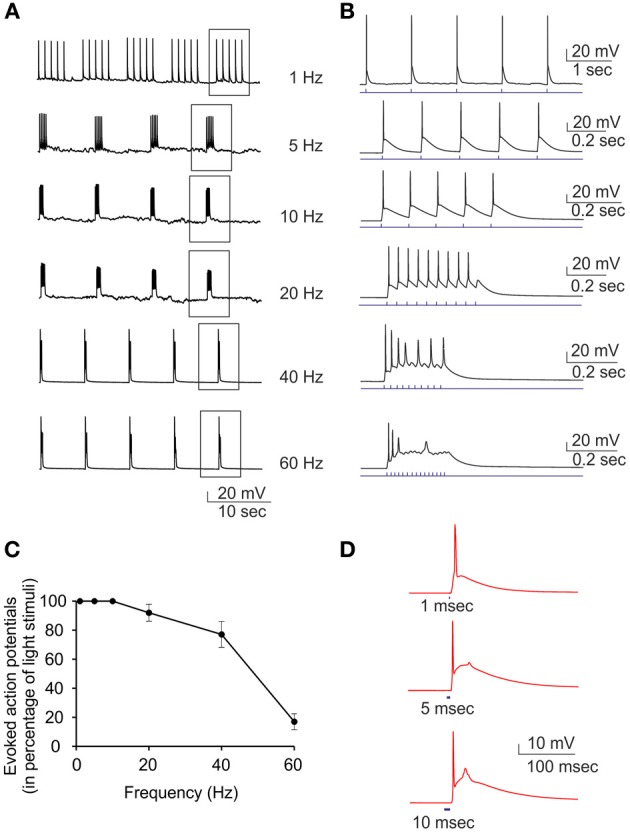
**Current-clamp characterization of ChR2/H134R-expressing neurons.** Single light pulses at 470 nm induce action potentials in neurons transduced with ChR2/H134R. **(A)** Representative traces showing the induction of action potentials by light pulses at increasing stimulation frequency (1–60 Hz). **(B)** Single trains boxed in panel **(A)** are shown in an expanded time scale. **(C)** Percent of successful action potential induced by light pulses as a function of the stimulation rate. Means ± s.e.m. are reported (*n* = 7). **(D)** Representative traces of single action potentials induced by light pulses of increasing duration (1, 5, and 10 ms). The afterdepolarization that limits the maximum frequency of light stimulation is clearly visible and increases with the pulse duration.

Current-clamp recordings were performed to demonstrate that the inward current observed in voltage-clamp recordings was able to induce action potential firing in ChR2/H134R-expressing neurons. Various light train stimuli from low (1 Hz) to high (60 Hz) frequency were administered to investigate their efficiency in triggering action potentials (Figures 3A,B). The ratio between the number of evoked action potentials and the number of light pulses was inversely dependent on the stimulation frequency and when the frequency reached 60 Hz, the percent of successful action potentials was reduced to 20% (Figure 3C). Moreover, when the duration of the light stimulus was changed from 1 to 10 ms, the probability to have an extra-action potential increased (Figure 3D). After these preliminary experiments, we passed to perform MEA recordings in our set-up to evaluate plastic responses by keeping the same conditions of light pulse duration, frequency and power and varying the stimulation protocols over time.

### Effects of tetanic optical stimulation on ChR2/H134R-expressing networks

**Figure 4:**
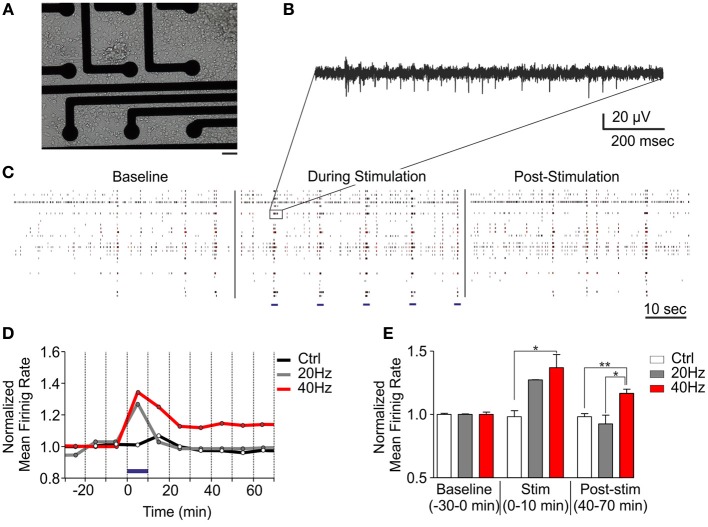
**Tetanic optical stimulation on ChR2/H134R-expressing networks induces long-term network plasticity.** Long-term changes in the neuronal network can be induced by modulating time and frequency of light stimulation. **(A)** 18 div neurons grown onto a MEA chip. Bar, 30 μm. **(B)** Representative extracellular recordings of action potential firing evoked by photostimulation on a single channel. **(C)** Raster plot representation of MEA recordings under basal conditions, during tetanic stimulation at 40 Hz and after photostimulation. Light stimuli are shown as blue horizontal bars in the middle panel. **(D)** Mean traces of the normalized mean firing rate (averaged in 10 min bins) during a long-term plasticity protocol consisting of 1 s stimulation at either 20 or 40 Hz (light pulse duration, 10 ms, blue horizontal bar) administered every 10 s for 10 min (blue horizontal bar). **(E)** Normalized mean firing rate under basal conditions (baseline), during photostimulation and after (time = 40–70 min) stimulation. Means ± s.e.m. are shown. YFP-infected control, 40 Hz, *n* = 5; ChR2/H134R-infected, 20 Hz, *n* = 3; ChR2/H134R-infected, 40 Hz, *n* = 4. ^*^p < 0.05, ^**^p < 0.01, Kruskal–Wallis test followed by the Dunn's multiple comparison test.

Synaptic plasticity has been well described since many years at the single cell level, but the impact of such functional and morphological modifications at a larger scale has remained largely unexplored. This was mainly due to the lack of appropriate electrophysiological techniques to perform long-lasting experiments on neuronal networks over the time needed by the network to implement the plastic short- and long-term events affecting single synaptic sites. To tackle this issue, we developed a fully integrated system to perform long-lasting extracellular electrophysiological recordings coupled with photostimulation and live optical imaging of primary neuronal networks grown onto MEA chips (Figure 4A). This system allowed us studying the impact of tetanic optical stimulation protocols on the entire network over timescales ranging from minutes to hours or even days (Figures 4B,C). Upon *in vitro* maturation, primary networks of hippocampal neurons grown onto MEA chips reveal a progressive development of rich spontaneous spiking and bursting activities that reflect the reverberant excitatory synaptic connections that progressively established during maturation (Chiappalone et al., 2009; Bologna et al., 2010; Zullo et al., 2012) (Figure 4B, baseline). We first applied tetanic protocols on ChR2/H134R-transduced neuronal cultures and evaluated the changes in network dynamics up to 1 h. Tetanic photostimulation was able to induce a multiple response on a single channel and also full network activation (Figures 4B,C). We used two stimulation protocols lasting 10 min consisting of 1 s train stimulation at either 20 or 40 Hz administered every 10 s (Figure 4D). We found that the 20 Hz train was not efficient to induce significant long-lasting changes in network dynamics either during or after the photo-stimulation period, whereas the 40 Hz train was effective in inducing long-term changes in the firing rate of the network (Figures 4D,E).

### Long-term effects of optical stimulation on ChR2/H134R-expressing networks

**Figure 5:**
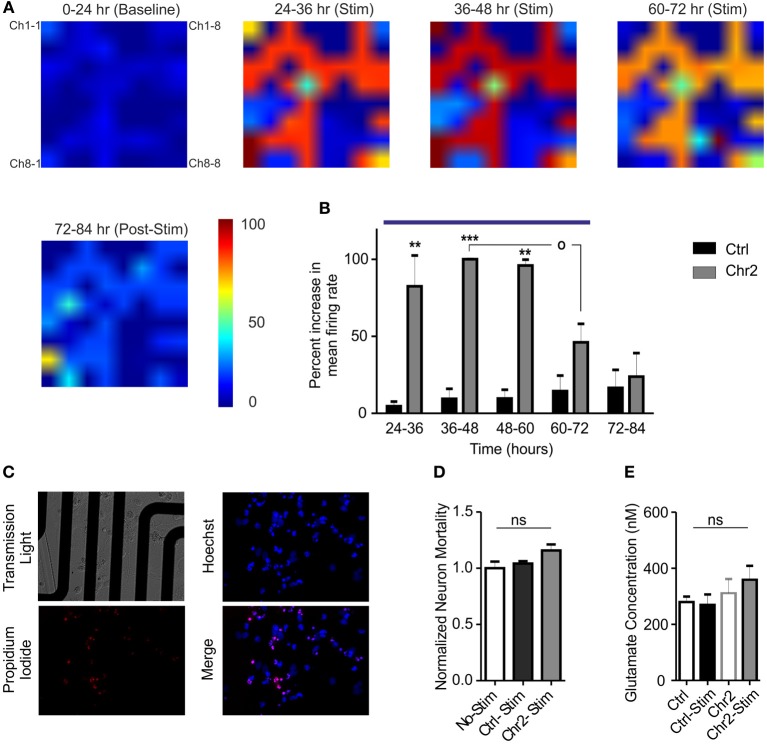
**Chronic stimulation with light pulse trains induces an adaptive response in hippocampal networks. (A)** Color map of the mean firing rate recorded on MEAs before (0–24 h epoch), during the administration of the photostimulation protocol (5 light pulses of 10 ms at 40 Hz every min for 48 h; 24–72 h epochs) and after stimulation (72–84 epoch). Color areas represent the normalized increases in the mean firing rate in the various epochs (see Materials and Methods and the look-up table reported on the right for pseudocolor coding). **(B)** The increase in the firing rate of ChR2/H134R-infected networks (gray bars; *n* = 5), measured in 12 h epochs, was normalized to the peak firing rate of ChR2/H134R-infected neurons over time. The normalized mean firing rate of stimulated control YFP-infected networks (black bars; *n* = 5) is shown for comparison. Networks expressing ChR2/H134R show neuronal adaptation to the chronic light stimulation (blue horizontal bar). Statistical analysis was performed using the Friedman's test for repeated measures followed by the Dunn's multiple comparison test. ^**^p < 0.01, ^***^p < 0.001 vs. control YFP-infected networks; °*p* < 0.05 vs. peak firing rate of ChR2/H134R-infected neurons. **(C)** Images described MEA cultures (upper left panel), Hoechst positive cells (blue, upper right panel), propidium iodide-positive cells (red, lower left panel) and merge image (lower right panel). **(D)** Normalized neuron mortality in non-stimulated YFP-infected cultures (white bars), YFP-infected cultures after chronic stimulation (black bars) and ChR2/H134R-infected cultures after stimulation (red bars). Means ± s.e.m. (*n* = 5 per experimental group) are shown. ns, not significant, Kuskal–Wallis test. **(E)** Mean ± s.e.m. (*n* = 5 per experimental group) glutamate concentrations analyzed before and after chronic stimulation in both YFP-infected control and ChR2/H134R-positive cultures. ns, Friedman's Two-Way ANOVA by Ranks.

Neural plasticity is a fundamental mechanism by which neural networks adapt to repeated external stimuli occurring over wide temporal windows. In this context, our system allows monitoring the entire network dynamics while performing naturally occurring protocols for prolonged amounts of time. Our OM-CSR system allowed us following the spatial evolution of extracellular network activity for long periods of time. We evaluated the occurrence of adaptive changes on the global firing activity while the network was subjected to a sustained external stimulation (Figure 5A). Control and ChR2/H134R-expressing cultures were recorded continuously for 24 h under basal conditions (0–24 h; baseline), subjected to repeated tetanic stimulation for 48 h (from 24 to 72 h; blue line in Figure 5B; see Materials and Methods) and followed for additional 12 h after stimulation (Figures 5A,B). The mean increase in the firing rate during photostimulation, normalized to the average peak response, showed that the culture responded by significantly increasing the activity several fold (average increase in the peak firing rate of ChR2/H134R-infected cultures with respect to baseline: 9.4 ± 3.5) during the first 36 h of stimulation (24–60 h; Figure 5B). However, during the last 12 h of photostimulation (60–72 h), the activity decreased with respect to the peak response (*p* < 0.05) and was not significantly different from the activity range of control cultures. Finally, when the sustained stimulation was ended, network activity fully returned to baseline levels, demonstrating that network dynamics could reset to the basal conditions.

The late decrease in firing could represent an adaptive response to the chronic stimulation or be the expression of network depopulation due to neuronal excitotoxic death. To verify that, we performed viability assays using propidium iodide and determination of extracellular glutamate concentrations immediately before and after the chronic stimulation protocol. No changes in neuron mortality were induced by photostimulation in either control or ChR2/H134R-infected cultures when compared to control, non-stimulated cultures (Figures 5C,D). Moreover, extracellular glutamate concentrations were similar in control and ChR2/H134R-infected cultures irrespective of the presence or absence of the sustained photostimulation protocol (Figure 5E) and the glutamate levels were much lower than the high μ M to mM concentrations responsible for excitotoxicity in cultured neurons (Berman and Murray, 1996; Ha et al., 2009). The absence of significant changes in either neuronal viability or extracellular glutamate concentrations rule out that the decrease in activity is due to neuronal death or distress and confirms the activation of adaptive changes in the network aimed at normalizing the activity in spite of the sustained state of hyperexcitation.

## Discussion

The functional and morphological properties of neuronal networks are strictly connected and the study of their relationships is an important aspect to understand the modular and hierarchical organization of the brain (Bassett et al., 2010). Although the complex and dense structure of the brain preserves invariant physiological features, *in vivo* experiments are difficult to evaluate because of the restricted accessibility of the entire neural circuits. *In vitro* experiments allow the control and investigation of the whole neuronal network, and offer a simple model to elucidate the interplay between morphological and functional properties (Shein-Idelson et al., 2011). The possibility to remotely control, and thus modify, the stimulation pattern without perturbing the environmental conditions of the networks, opens the possibility to study the plastic effects of distinct protocols over short and long time windows (Potter and Demarse, 2001).

The study of long-term adaptive changes in primary networks is intrinsically difficult, as stimulation and recording protocols typically require withdrawal of the cultures from the incubator and stimulation/recordings at the set-up stage generally at room temperature (Bologna et al., 2010). For this reasons, the plastic changes occurring in the timescale of hours or days are difficult to follow. In addition, electrical stimulation cannot be spatially controlled, or cell-type specifically targeted (Wallach et al., 2011). Long-lasting electrical stimulation protocols, besides generating large recording artifacts, can be performed only from few electrodes and therefore the network dynamics can be altered only locally.

We show here that we were able to modulate the entire network dynamics by using a simple and low cost setup, which allowed us to optically stimulate the entire network and record thereof. The OM-CSR system can be easily adapted to perform site-specific stimulation by shaping the light and/or targeting ChR2 expression to specific neuronal populations (Knopfel et al., 2010; Fenno et al., 2011; Mei and Zhang, 2012). The use of optogenetics turned out to be very efficient to perform stimulation protocols with high flexibility and high efficiency in eliciting action potential firing widespread in the network. A common limitation for performing electrophysiological recordings while neurons are kept in a CO_2_ incubator is the quasi-saturating aqueous vapor that, in the long run, degrades the electrode surfaces, alters electronic wiring and optical component properties, no matter how protected and isolated they are. We eliminated the humidity control in the incubator environment and, at the same time, prevented evaporation of the cell culture medium. We found that neurons could survive under these conditions for over 3 weeks, developing normally and preserving their passive and active electrophysiological properties. With such an integrated system, we could apply distinct stimulation protocols on the very same neural network to directly compare their efficiency. While stimulation at 20 Hz did not alter the network mean firing rate, stimulation at 40 Hz significantly increased spontaneous activity and the effect lasted up to 1 h after light exposure. Notably, our system allowed to reproducibly analyze the two protocols on the very same sample over time, excluding the variability among independent *in vitro* random networks. When chronic stimulation was applied, the activity of the ChR2/H134R-infected networks underwent adaptive changes that were apparent 48–60 h after the onset of the light stimulation and that are likely to reflect adaptive modifications of intrinsic excitability and synaptic strength at single neuronal units (Turrigiano, 2012).

One of the challenging applications of optogenetics is the possibility to dissect and treat neurological disorders. Although the development of implantable optical systems to stimulate single neurons in the brain still requires further technological improvements (PiyawattanametHa et al., 2009), it is fundamental to screen chronic and stable light modulating protocols for correcting brain dysfunctions and develop well-targeted viral expression systems. By studying the potential toxic effects and the neural network responses to chronic optical stimulation, this set-up may allow refining stimulation protocols to be used for the development of bidirectional neuro-prostheses (O'Doherty et al., 2011).

Optogenetics has opened a new avenue for exploring the functions of complex neural networks in the normal brain, as well as for dissecting the pathogenesis of various neuropsychiatric conditions and proposing new potential curative approaches based on photostimulation (Kokaia and Sorensen, 2011; Tye and Deisseroth, 2012). Restoration of dopamine-related movement dysfunction in parkinsonian animals, relief of anxiety, silencing of seizures in epileptic animals or correction of blindness in models of *Retinitis pigmentosa* are just few examples of the potential applications. In this perspective, our system allows the long-term screening of optogenetic methods, not only to assess the effects of chronic photostimulation protocols and to dissect the network response to these stimulation protocols, but also to mimic *in vitro* deep brain stimulation protocols that have been demonstrated to be effective in treating drug-resistant forms of Parkinson's disease and/or depression.

In conclusion, the OM-CSR system described here allows performing these fundamental studies at the network level and may contribute to a better understanding of the principles of long-term modulation of neural networks under physiological conditions and in experimental models of neurological diseases.

### Conflict of interest statement

The authors declare that the research was conducted in the absence of any commercial or financial relationships that could be construed as a potential conflict of interest.

## References

[B1] Abbott L. F. Regehr W. G. (2004). Synaptic computation. Nature 431, 796–803 10.1038/nature03010 15483601

[B2] Baldelli P. Fassio A. Valtorta F. Benfenati F. (2007). Lack of synapsin I reduces the readily releasable pool of synaptic vesicles at central inhibitory synapses. J. Neurosci. 27, 13520–13531 10.1523/JNEUROSCI.3151-07.2007 18057210 PMC6673103

[B3] Bassett D. S. Greenfield D. L. Meyer-Lindenberg A. Weinberger D. R. Moore S. W. Bullmore E. T. (2010). Efficient physical embedding of topologically complex information processing networks in brains and computer circuits. PLoS Comput. Biol. 6:e1000748 10.1371/journal.pcbi.1000748 20421990 PMC2858671

[B4] Berman F. W. Murray T. F. (1996). Characterization of glutamate toxicity in cultured rat cerebellar granule neurons at reduced temperature. J. Biochem. Toxicol. 11, 111–119 9029269 10.1002/(SICI)1522-7146(1996)11:3<111::AID-JBT2>3.0.CO;2-N

[B5] Biffi E. Regalia G. Ghezzi D. De Ceglia R. Menegon A. Ferrigno G. (2012). A novel environmental chamber for neuronal network multisite recordings. Biotechnol. Bioeng. 109, 2553–2566 10.1002/bit.24526 22510865

[B6] Blau A. Neumann T. Ziegler C. Benfenati F. (2009). Replica-moulded polydimethylsiloxane culture vessel lids attenuate osmotic drift in long-term cell cultures. J. Biosci. 34, 59–69 10.1007/s12038-009-0009-3 19430119

[B7] Blau A. W. Ziegler C. M. (2001). Prototype of a novel autonomous perfusion chamber for long-term culturing and *in situ* investigation of various cell types. J. Biochem. Biophys. Methods 50, 15–27 10.1016/S0165-022X(01)00163-4 11714508

[B8] Bologna L. L. Nieus T. Tedesco M. Chiappalone M. Benfenati F. Martinoia S. (2010). Low-frequency stimulation enhances burst activity in cortical cultures during development. Neuroscience 165, 692–704 10.1016/j.neuroscience.2009.11.018 19922773

[B9] Caroni P. Donato F. Muller D. (2012). Structural plasticity upon learning: regulation and functions. Nat. Rev. Neurosci. 13, 478–490 10.1038/nrn3258 22714019

[B10] Chang M. C. Park J. M. Pelkey K. A. Grabenstatter H. L. Xu D. Linden D. J. (2010). Narp regulates homeostatic scaling of excitatory synapses on parvalbumin-expressing interneurons. Nat. Neurosci. 13, 1090–1097 10.1038/nn.2621 20729843 PMC2949072

[B11] Chiappalone M. Casagrande S. Tedesco M. Valtorta F. Baldelli P. Martinoia S. (2009). Opposite changes in glutamatergic and GABAergic transmission underlie the diffuse hyperexcitability of synapsin I-deficient cortical networks. Cereb. Cortex 19, 1422–1439 10.1093/cercor/bhn182 19020204

[B12] Chiappalone M. Massobrio P. Martinoia S. (2008). Network plasticity in cortical assemblies. Eur. J. Neurosci. 28, 221–237 10.1111/j.1460-9568.2008.06259.x 18662344

[B13] Daoudal G. Debanne D. (2003). Long-term plasticity of intrinsic excitability: learning rules and mechanisms. Learn. Mem. 10, 456–465 10.1101/lm.64103 14657257

[B14] De Palma M. Naldini L. (2002). Transduction of a gene expression cassette using advanced generation lentiviral vectors. Methods Enzymol. 346, 514–529 10.1016/S0076-687946074-0 11883088

[B15] Deng P. Y. Klyachko V. A. (2011). The diverse functions of short-term plasticity components in synaptic computations. Commun. Integr. Biol. 4, 543–548 22046457 10.4161/cib.4.5.15870 PMC3204123

[B16] Dranias M. R. Ju H. Rajaram E. Vandongen A. M. (2013). Short-term memory in networks of dissociated cortical neurons. J. Neurosci. 33, 1940–1953 10.1523/JNEUROSCI.2718-12.2013 23365233 PMC6619106

[B17] Feldman D. E. (2012). The spike-timing dependence of plasticity. Neuron 75, 556–571 10.1016/j.neuron.2012.08.001 22920249 PMC3431193

[B18] Fenno L. Yizhar O. Deisseroth K. (2011). The development and application of optogenetics. Annu. Rev. Neurosci. 34, 389–412 10.1146/annurev-neuro-061010-113817 21692661 PMC6699620

[B19] Fioravante D. Regehr W. G. (2011). Short-term forms of presynaptic plasticity. Curr. Opin. Neurobiol. 21, 269–274 10.1016/j.conb.2011.02.003 21353526 PMC3599780

[B20] Frega M. Pasquale V. Tedesco M. Marcoli M. Contestabile A. Nanni M. (2012). Cortical cultures coupled to micro-electrode arrays: a novel approach to perform *in vitro* excitotoxicity testing. Neurotoxicol. Teratol. 34, 116–127 10.1016/j.ntt.2011.08.001 21856414

[B21] Goold C. P. Nicoll R. A. (2010). Single-cell optogenetic excitation drives homeostatic synaptic depression. Neuron 68, 512–528 10.1016/j.neuron.2010.09.020 21040851 PMC3111089

[B22] Gross G. W. Schwalm F. U. (1994). A closed flow chamber for long-term multichannel recording and optical monitoring. J. Neurosci. Methods 52, 73–85 10.1016/0165-027090059-0 8090021

[B23] Grubb M. S. Burrone J. (2010). Activity-dependent relocation of the axon initial segment fine-tunes neuronal excitability. Nature 465, 1070–1074 10.1038/nature09160 20543823 PMC3196626

[B24] Gunaydin L. A. Yizhar O. Berndt A. Sohal V. S. Deisseroth K. Hegemann P. (2010). Ultrafast optogenetic control. Nat. Neurosci. 13, 387–392 10.1038/nn.2495 20081849

[B25] Ha J. S. Lee C. S. Maeng J. S. Kwon K. S. Park S. S. (2009). Chronic glutamate toxicity in mouse cortical neuron culture. Brain Res. 1273, 138–143 10.1016/j.brainres.2009.03.050 19344697

[B26] Kim S. Jung U. Baek J. Kang S. Kim J. (2012). Simultaneous measurement of neural spike recordings and multi-photon calcium imaging in neuroblastoma cells. Sensors 12, 15281–15291 10.3390/s121115281 23202210 PMC3522963

[B27] Knopfel T. Lin M. Z. Levskaya A. Tian L. Lin J. Y. Boyden E. S. (2010). Toward the second generation of optogenetic tools. J. Neurosci. 30, 14998–15004 10.1523/JNEUROSCI.4190-10.2010 21068304 PMC2997431

[B28] Kokaia M. Sorensen A. T. (2011). The treatment of neurological diseases under a new light: the importance of optogenetics. Drugs Today (Barc) 47, 53–62 10.1358/dot.2011.47.1.1543306 21373649

[B29] Lau C. G. Murthy V. N. (2012). Activity-dependent regulation of inhibition via GAD67. J. Neurosci. 32, 8521–8531 10.1523/JNEUROSCI.1245-12.2012 22723692 PMC3388776

[B30] Lin J. Y. (2011). A user's guide to channelrhodopsin variants: features, limitations and future developments. Exp. Physiol. 96, 19–25 10.1113/expphysiol.2009.051961 20621963 PMC2995811

[B31] Lin J. Y. Lin M. Z. Steinbach P. Tsien R. Y. (2009). Characterization of engineered channelrhodopsin variants with improved properties and kinetics. Biophys. J. 96, 1803–1814 10.1016/j.bpj.2008.11.034 19254539 PMC2717302

[B32] Mei Y. Zhang F. (2012). Molecular tools and approaches for optogenetics. Biol. Psychiatry 71, 1033–1038 10.1016/j.biopsych.2012.02.019 22480664 PMC3529099

[B33] O'Doherty J. E. Lebedev M. A. Ifft P. J. Zhuang K. Z. Shokur S. Bleuler H. (2011). Active tactile exploration using a brain-machine-brain interface. Nature 479, 228–231 10.1038/nature10489 21976021 PMC3236080

[B34] Pancrazio J. J. Gray S. A. Shubin Y. S. Kulagina N. Cuttino D. S. Shaffer K. M. (2003). A portable microelectrode array recording system incorporating cultured neuronal networks for neurotoxin detection. Biosens. Bioelectron. 18, 1339–1347 10.1016/S0956-566300092-7 12896834

[B35] Piyawattanametha W. Cocker E. D. Burns L. D. Barretto R. P. Jung J. C. Ra H. (2009). *In vivo* brain imaging using a portable 2.9 g two-photon microscope based on a microelectromechanical systems scanning mirror. Opt. Lett. 34, 2309–2311 10.1364/OL.34.002309 19649080 PMC2826365

[B36] Potter S. M. Demarse T. B. (2001). A new approach to neural cell culture for long-term studies. J. Neurosci. Methods 110, 17–24 10.1016/S0165-027000412-5 11564520

[B37] Remme M. W. Wadman W. J. (2012). Homeostatic scaling of excitability in recurrent neural networks. PLoS Comput. Biol. 8:e1002494 10.1371/journal.pcbi.1002494 22570604 PMC3342932

[B38] Shein-Idelson M. Ben-Jacob E. Hanein Y. (2011). Engineered neuronal circuits: a new platform for studying the role of modular topology. Front. Neuroeng. 4:10 10.3389/fneng.2011.00010 21991254 PMC3180629

[B39] Shin S. M. Zhang N. Hansen J. Gerges N. Z. Pak D. T. Sheng M. (2012). GKAP orchestrates activity-dependent postsynaptic protein remodeling and homeostatic scaling. Nat. Neurosci. 15, 1655–1666 10.1038/nn.3259 23143515 PMC3804128

[B40] Takahashi H. Sakurai T. Sakai H. Bakkum D. J. Suzurikawa J. Kanzaki R. (2012). Light-addressed single-neuron stimulation in dissociated neuronal cultures with sparse expression of ChR2. Biosystems 107, 106–112 10.1016/j.biosystems.2011.10.002 22019848

[B41] Tessadori J. Bisio M. Martinoia S. Chiappalone M. (2012). Modular neuronal assemblies embodied in a closed-loop environment: toward future integration of brains and machines. Front. Neural Circuits 6:99 10.3389/fncir.2012.00099 23248586 PMC3520178

[B42] Turrigiano G. (2012). Homeostatic synaptic plasticity: local and global mechanisms for stabilizing neuronal function. Cold Spring Harb. Perspect. Biol. 4:a005736 10.1101/cshperspect.a005736 22086977 PMC3249629

[B43] Turrigiano G. G. (2008). The self-tuning neuron: synaptic scaling of excitatory synapses. Cell 135, 422–435 10.1016/j.cell.2008.10.008 18984155 PMC2834419

[B44] Tye K. M. Deisseroth K. (2012). Optogenetic investigation of neural circuits underlying brain disease in animal models. Nat. Rev. Neurosci. 13, 251–266 10.1038/nrn3171 22430017 PMC6682316

[B45] Wallach A. Eytan D. Gal A. Zrenner C. Marom S. (2011). Neuronal response clamp. Front. Neuroeng. 4:3 10.3389/fneng.2011.00003 21519391 PMC3078750

[B46] Yang H. Shew W. L. Roy R. Plenz D. (2012). Maximal variability of phase synchrony in cortical networks with neuronal avalanches. J. Neurosci. 32, 1061–1072 10.1523/JNEUROSCI.2771-11.2012 22262904 PMC3319677

[B47] Zullo L. Chiappalone M. Martinoia S. Benfenati F. (2012). A spike-based grammar underlies directional modification in network connectivity: effect on bursting activity and implications for bio-hybrids systems. PLoS ONE 7:e49299 10.1371/journal.pone.0049299 23145147 PMC3493547

